# Adhesion as a Focus in Trichoderma–Root Interactions

**DOI:** 10.3390/jof8040372

**Published:** 2022-04-06

**Authors:** James T. Taylor, Rebekka Harting, Samer Shalaby, Charles M. Kenerley, Gerhard H. Braus, Benjamin A. Horwitz

**Affiliations:** 1 Department of Plant Pathology and Microbiology, Texas A&M University, College Station, TX 77843, USA; jimtaylor@aggienetwork.com (J.T.T.); c-kenerley@tamu.edu (C.M.K.); 2 Department of Molecular Microbiology and Genetics, Institute of Microbiology and Genetics, University of Göttingen, 37077 Göttingen, Germany; rhartin@gwdg.de (R.H.); gbraus@gwdg.de (G.H.B.); 3 Faculty of Biology, Technion—Israel Institute of Technology, Haifa 3200000, Israel; sam.shalaby@gmail.com

**Keywords:** Trichoderma, root, rhizosphere, mutualism, adhesion, adhesin, fungal, transcriptional

## Abstract

Fungal spores, germlings, and mycelia adhere to substrates, including host tissues. The adhesive forces depend on the substrate and on the adhesins, the fungal cell surface proteins. Attachment is often a prerequisite for the invasion of the host, hence its importance. Adhesion visibly precedes colonization of root surfaces and outer cortex layers, but little is known about the molecular details. We propose that by starting from what is already known from other fungi, including yeast and other filamentous pathogens and symbionts, the mechanism and function of Trichoderma adhesion will become accessible. There is a sequence, and perhaps functional, homology to other rhizosphere-competent Sordariomycetes. Specifically, *Verticillium dahliae* is a soil-borne pathogen that establishes itself in the xylem and causes destructive wilt disease. Metarhizium species are best-known as insect pathogens with biocontrol potential, but they also colonize roots. Verticillium orthologs of the yeast Flo8 transcription factor, Som1, and several other relevant genes are already under study for their roles in adhesion. Metarhizium encodes relevant adhesins. *Trichoderma virens* encodes homologs of Som1, as well as adhesin candidates. These genes should provide exciting leads toward the first step in the establishment of beneficial interactions with roots in the rhizosphere.

## 1. Introduction

Interaction of Trichoderma with roots primes plants for a stronger immune response to pathogens, and can also promote growth [1,2,3,4]. Some interactions occur from a distance, through the emission of volatile organic compounds (VOCs) and secretion of diffusible factors, while other modes depend on colonization by fungal mycelia. Trichoderma colonizes, at first, between living plant cells, resembling the early steps in an attack by soilborne pathogens. The fungus, however, is restricted to the outer root tissue layers by plant defenses, for example, salicylic acid (SA) and the SA-mediated pathway [5]. To initiate colonization, conidial germ tubes or hyphae growing near and toward the root in the rhizosphere apparently adhere to the root epidermis. Adhesion has been documented for soilborne pathogens, which adhere before they begin to damage the host root tissue [6,7]. Although the plant tissues remain intact at first, it is still possible that the surface-surface interaction is, perhaps to a varying extent, host plant-specific. Trichoderma has a very wide plant host range, an important factor in the choice of these fungi for biocontrol. The molecular basis for initial recognition (if it is indeed specific) and adherence is, therefore, of great interest. There is little information about this topic, and here we will address how the molecular mechanisms could be studied.

Adhesion is a fundamental part of fungal lifestyles: It is an initial step in disease (and symbiosis), developmental cascades, and biofilm formation. Fungal germ tubes can also adhere to each other immediately following emergence from the spore and preceding cell–cell fusion. Fungi residents in the rhizosphere could be carried to the roots by the bulk flow of water carrying spores. Alternatively, and better-documented, germ tubes or mycelia reach the root surface by hyphal growth over short distances. Growth is guided by chemotropism toward root-derived signals [8,9,10]. Contact between the fungal and plant cell surfaces produces adhesive forces, stabilizing the interaction and ultimately allowing penetration into or between plant cells. A green fluorescent protein (Gfp)-expressing strain permitted closely resolved visualization of the early stages of infection of tomato roots by *Fusarium oxysporum f.* sp. *radicis-lycopersici* [7]. Among the first applications of Gfp expression to filamentous ascomycetes, these experiments clearly visualized the interaction between the pathogen and tomato root hairs and the epidermal surface prior to more extensive colonization and ingress. Bean root exudates modified the transcriptomic program of *Metarhizium anisopliae*, consistent with the dual niche of this species as a pathogen of insects and a rhizosphere-competent partner of roots [11]. Among the upregulated genes, adhesin Mad2 specifically mediates adhesion to plant surfaces, as shown by loss of adhesion in deletion mutants; expression of Mad2 in yeast is indeed sufficient to make *S. cerevisiae* adhere to onion epidermis. Another adhesin, Mad1, mediates the adhesion to insect cuticles [12]. *M. anisopliae* adhesin functional expression in yeast, aside from its use as an assay [7,8], implies conservation of the mechanism from yeast to filamentous fungi. This hypothesis also provided the context for the study of Flo8-related transcription factors in filamentous ascomycetes. *Verticillium dahliae* causes destructive wilt disease. Verticillium infections start at the plant roots. Resting structures (microsclerotia) germinate and the fungus grows toward the root, which it penetrates to colonize the host plant. The attachment of the hyphae to the roots might require adhesion processes. The exact mechanisms are not yet understood but several proteins have been described to be involved in the process or its regulation. Examples include the transmembrane mucin Msb, the *V. dahliae* Flo8 homolog Som1, Verticillium transcriptional activator of adhesion 3 (Vta3), the MADS-box transcription factor Mcm1, as well as the small GTPase Rac1 and the p21-activated kinase Cla4 [13,14,15,16]. Deletion of either of the corresponding genes did not only affect the adhesion of the fungus to the roots and/or its initial colonization, but also resulted in reduced pathogenicity and phenotypical alterations, as, for example, impaired conidiation and/or resting structure formation. Another process that involves adhesion is the formation of resting structures (microsclerotia), which ensure the survival of the fungus until a suitable host is present. Vdh1 is a hydrophobin, which was shown to be important for this process [17,18]. There seems to be a complex transcriptional network that controls adhesion, root colonization, further plant colonization, virulence, and fungal development (see below).

Mycorrhizal symbionts also have an attachment stage. Following proliferation and branching triggered by plant strigolactone signals [19], the initial contact of arbuscular mycorrhizal fungi (AFM) with roots leads to the development of hyphopodia, which adhere to the epidermis [20,21]. Molecular details of adhesins and their expression are apparently unknown, as for ectomycorrhizae, so that the situation even for these critically important symbionts is like that for Trichoderma.

## 2. Adhesion of Trichoderma

**Figure 1:**
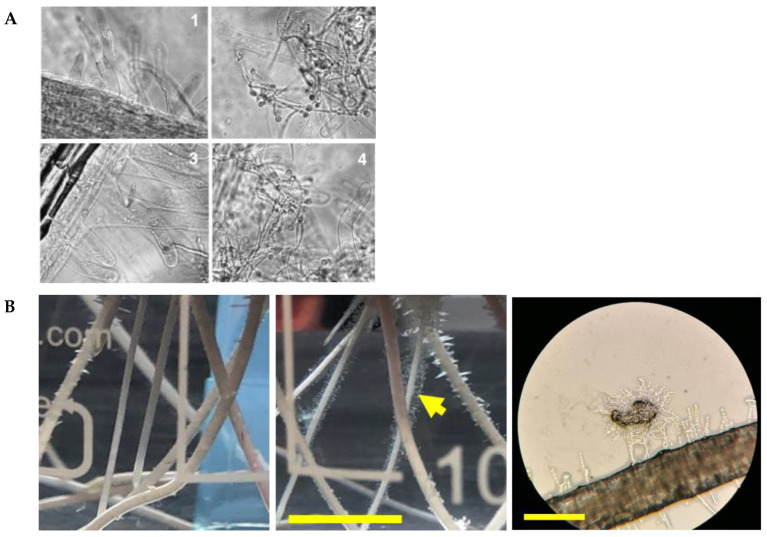
Adhesion of germinating Trichoderma conidia. (**A**) Adherence of *T. asperellum* to cucumber roots in hydroponics, and role of a hydrophobin, TasHyd1 (image from [22], *Molecular Plant Pathology*, Wiley, open access). The roots were imaged 48 h post-inoculation: 1, non-inoculated control; 2, wild type; 3, TasHyd1 deletion mutant; 4, complemented strain. The large cells are root hairs. (**B**) *Trichoderma virens* germlings adhering in hydroponic culture. Left, germlings of *T. virens* adhering to maize roots in hydroponic culture: spores germinated 16 h to inoculation time, incubation with roots on a rotary shaker for 3 h, scale bar 10 mm; note decoration of root hairs with adhering germlings (arrow) (Horwitz, unpublished). Right, germlings accumulated on root hairs; scale bar approximately 250 μm (Taylor, unpublished).

Germlings and mycelia of Trichoderma adhere to plant roots. This first became apparent in studies where the fungus interacted with roots in hydroponic culture [22,23] (Figure 1). Deletion experiments showed that TasHyd1 is necessary for adhesion [22] (Figure 1A). The extent to which attachment itself (or adhesion-independent functions of the hydrophobin) is needed for the fungus to prime the plant’s immune responses has apparently not yet been tested. Loss of attachment does correlate with decreased colonization; however, the extent of colonization is not necessarily closely linked to the extent of immune priming.

### 2.1. Molecular Basis of Adhesion

The most detailed knowledge of fungal adhesins and their regulation comes from pathogenic or model yeasts. Classic yeast genetics experiments showed that many laboratory strains have lost the potential for adhesion and flocculation because of the loss of regulatory genes, whereas “Sigma” yeast strains, as well as natural isolates, normally adhere. In the Sigma background, adhesion depends on the filamentation MAPK (fMAPK) pathway, and adherence can be restored in laboratory strains by the dominant gene encoding transcription factor Flo8 [24,25,26]. The key to understanding the regulatory hierarchy may be the large promoter of adhesin gene Flo11, which among others is activated by Flo8 [27]. Differential regulation of targets downstream of Flo8 was shown in yeast [28]. The deletion of a single adhesin gene, EPA1, nearly eliminated the adherence of *Candida glabrata* to human epithelial cells. The *FLO* genes of *Saccharomyces cerevisiae* encode cell wall-associated adhesins. *FLO1* and *FLO11* promote flocculation in liquid as well as substrate adhesion. Flo1 protein is required for both adhesion and flocculation, whereas Flo11 is responsible for the initial surface adhesion and cell–cell interaction. Both genes are activated by the transcription factor Flo8, which is controlled by the cAMP/PKA signal transduction pathway [27].

Our knowledge of filamentous fungi, including plant pathogens, is still limited at the molecular level. Adhesion of conidia, often before germination, to plant leaves is critical for establishing disease. Defects in penetration or colonization steps block the ability of fungal pathogens to infect plants. Adhesive proteins for penetration of the root surface are required at several stages during the host–parasite interaction. The CAP20 protein is formed during appressoria formation and is necessary for adhesion and virulence of the plant pathogenic fungus *Colletotrichum gloeosporioides* [29]. The fasciclin domain protein Fas1 is crucial for appressorial turgor in *Magnaporthe oryzae* [30]. Filamentous fungal orthologs of Flo8 (SomA, Som1) and other regulators isolated from screens in yeast of *Verticillium longisporum* cDNA clones, are indeed sufficient to induce adhesion when expressed in a non-adherent yeast background [14,31,32].

### 2.2. Adhesins

**Figure 2:**
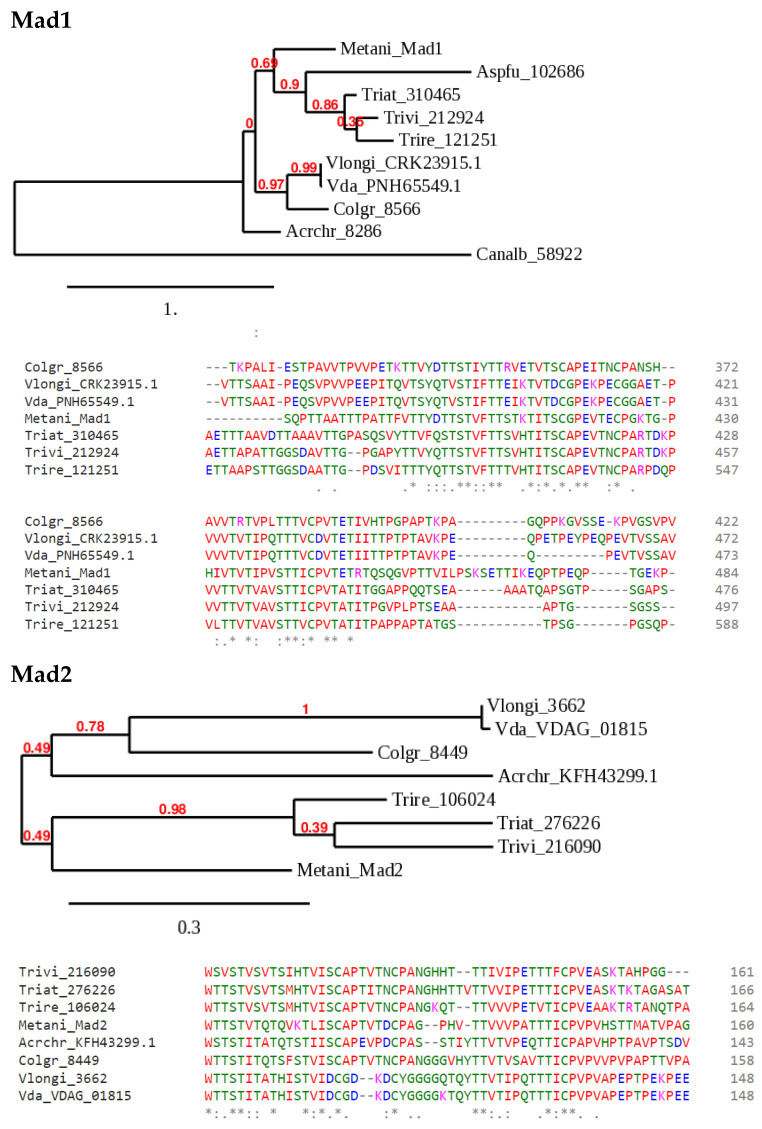
Sample phylogenies of candidate Als group adhesins in Trichoderma species. *Metarhizium anisopliae* Mad1 and Mad2 [12] were used to search Trichoderma and a few selected ascomycete databases at JGI Mycocosm [42] or the NCBI protein database by BLASTP. The available, computer-generated annotations do not correspond to adhesins, and these genes have not been manually annotated. Annotations by simple numbers are JGI protein IDs, those beginning with letters are from NCBI, and VDAG_01815 is a *V. dahliae* database accession corresponding to JGI ID 6481. The species abbreviations are: Metani, *Metarhizium anisopliae*; Aspfu, *Aspergillus fumigatus*; Vda, *Verticillium dahliae*; Vlongi, *Verticillium longisporum*; Colgr, *Colletotrichum graminearum*; Acrchr, *Acremonium chrysogenum*; Canalb, *Candida albicans*; Trivi, *Trichoderma virens*; Triat, *Trichoderma atroviride*; Trire, *Trichoderma reesei*. The trees were generated at http://phylogeny.fr ([43], accessed on 30 March 2022; the numbers in red at each node are simulated bootstrap values). For the alignments shown below the trees, the sequences were aligned using CLUSTAL Omega (https://www.ebi.ac.uk/Tools/msa/clustalo/, accessed on 25 March 2022). A portion of each of the two alignments, corresponding to part of a threonine-rich region identified in all these sequences by PROSITE (https://prosite.expasy.org/scanprosite/, accessed on 25 March 2022), is shown below each tree diagram (color coding indicates amino acid class; the symbols *: below alignments indicate identity/similarity). In the Mad1 phylogeny, *A. chrysogenum* and *C. albicans* are relatively distant, aligned with large gaps, and were excluded from the alignment shown. Likewise, a region of *C. graminicola* Mad2 that created a gap in the alignment of all the other sequences was removed manually before making the alignment shown. The trees were generated from the full alignments of all the complete sets of protein sequences.

Proteins that are anchored to the fungal cell and adhere to other cells, substrates, or host tissues create adhesive forces. These, generally glycosylated, are collectively known as adhesins. Most adhesins comprise three modules: an exposed N-terminal domain that binds the target ligand, a middle segment characterized by serine and threonine-rich repeats, and a C-terminal GPI anchor domain. The targets can be glycans or peptides. In the context of Trichoderma–root interactions (as for other symbionts and plant pathogens), plant cell walls are clearly rich in possible binding targets. Adhesins encoded by pathogens and symbionts are under selection for adaptation to the host [33], as shown for *Candida glabrata* [34]. Metarhizium, having evolved as an insect pathogen and plant symbiont, has some similarity to Trichoderma (mycotroph and plant symbiont), and adaptation to interaction with plants is an evolutionary force acting on Metarhizium adhesins [35]. Fungal adhesins are encoded by three main gene families: PA14-type, Flo11-type, and Als-type [33,36,37]. The potential to form functional amyloid structures [38] has been found in Als (Agglutinin-like sequence) type adhesins [39]. Trichoderma genomes encode adhesin candidates; however, these have not been characterized by genetic experiments. Gene deletion of the *T. asperellum* hydrophobin TasHyd1 (Figure 1) indeed prevented adhesion, suggesting that this will be a promising approach. Starting with the Metarhizium Als family adhesins Mad1 and Mad2, we identified the best hits by BLASTP searches, and generated two sample phylogenies, to illustrate how analysis of Trichoderma adhesins can begin (Figure 2). These sequences contain a region with a repeat structure characteristic of this adhesin class. Another signature is a threonine-rich region, part of which shows obvious consensus (Figure 2, alignments). Furthermore, a predictor for potential GPI lipid modification sites in fungi (https://mendel.imp.ac.at/gpi/fungi_server.html, accessed on 25 March 2022 [39]) detected, with high confidence, a GPI anchor in all the Mad1 and Mad2 sequences. Together, these signatures lend support to the identification of these genes as Trichoderma adhesin candidates. The Mad2 orthologs, because of their documented role in Metarhizium–root interactions as mentioned above, might be a first choice for reverse genetics. The approach, indeed, can be widened to encompass all three adhesin superfamilies. Identification of new candidates is not trivial, depending on the taxonomic distance: the novel *Ustilago maydis* adhesin Lep1 [40], for example, yielded no BLASTP hits to the *T. virens* predicted proteome. Conversely, BLASTP hits with Metarhizium Mad1 and Mad2 were restricted to ascomycete genomes with the most confidence for closely related Sordariomycetes. Without overgeneralizing, it may be that the regulatory proteins are more conserved, as compared to the regulated “output” proteins (adhesins). Other approaches, for example co-regulation with transcript clusters expressed in interaction with plant roots, are therefore needed to identify relevant adhesins. In *T. virens*, a gene with a GLEYA domain (a lectin-like domain which defines a Flo adhesin subtype in yeasts [36]), ID 10277, is expressed both in axenic culture and in interaction with maize roots [41] (see Section 2.3.2). The expression of two *V. dahliae* adhesin candidates, Fas1 and Wsc1, de pends on the transcriptional activator of adhesion Vta2 [32]. Searches by BLASTP on the *T. virens* database with *V. dahliae* Fas1 identified ID 172511 at 86% coverage, 6 × 10^−33^, and with Wsc1 ID 79838 at 53% coverage, 8 × 10^−18^. The respective domains are detected by Pfam in the *T. virens* homologs. The construction of the deletion and overexpression mutants in these adhesin gene candidates can assign functional roles in Trichoderma species.

### 2.3. Regulation of the Adhesion Program at the Transcriptional Level

#### 2.3.1. Trichoderma Flo8 Orthologs

**Figure 3:**
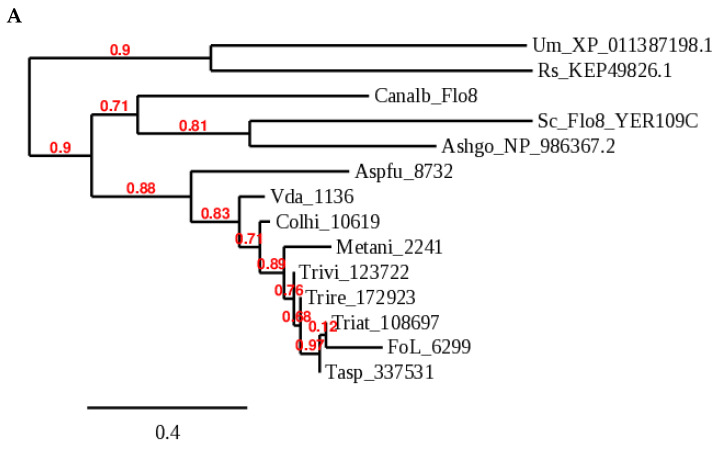

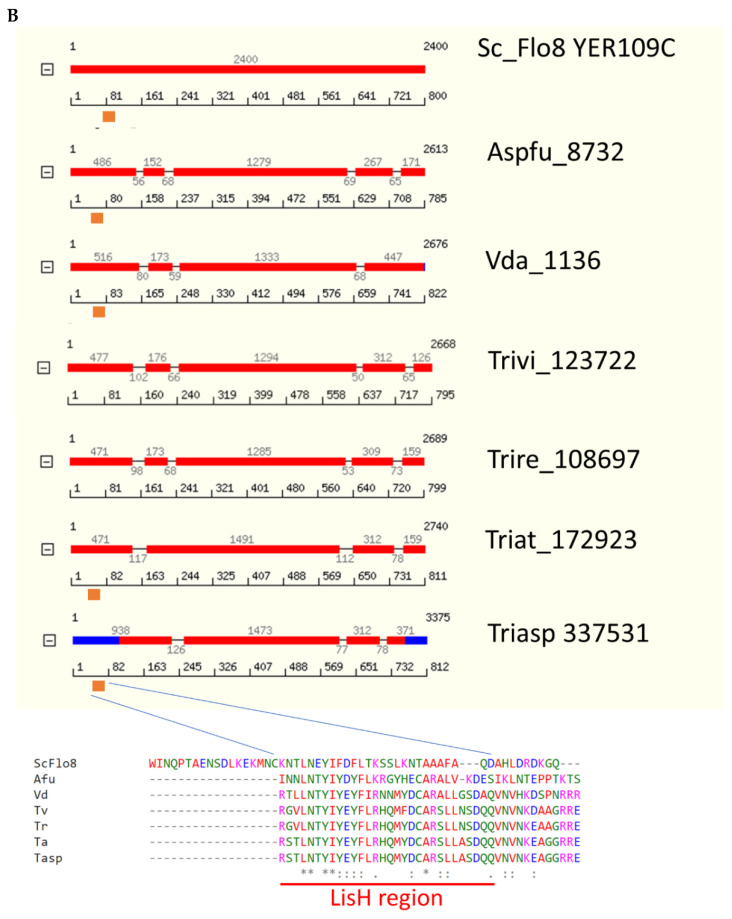
Som1/Flo8 orthologs of several Trichoderma species. (**A**) Phylogeny generated at Phylogeny.fr: Phylogeny Analysis [43]. (**B**) Protein models. Predicted protein sequences and intron structures are from JGI Mycocosm (https://mycocosm.jgi.doe.gov/mycocosm/home, accessed on 19 December 2021; [42,44,45]). Red bars indicate coding sequence and blue bars, 5’ and 3’ untranslated regions. Species abbreviations in addition to those in Figure 2 are: Sc, *Saccharomyces cerevisiae*; Tasp, *Trichoderma asperellum*; FoL, *Fusarium oxysporum f.* sp. *lycopersici 4287*; Ashgo, *Eremothecium (Ashbya) gossypii*; Um, *Ustilago maydis*; Rs, *Rhizoctonia solani*; Colhi, *Colletotrichum higginsianum*. The numbers are JGI database protein IDs or NCBI accessions.

We already know that the Flo8 transcription factor (TF) is functionally conserved from yeast to *A. fumigatus* (SomA) and *V. dahliae* (Som1). TFs provide a starting point to identify an entire regulon; furthermore, specific adhesins could be linked to host-specific phenotypes, as in the case of Mad1,2 of Metarhizium as discussed above. We searched the *T. virens* genome with Som1 from the related ascomycete, *V. dahliae*, and identified a single candidate, named TvSom1, at 1.68 × 10^−156^, with 69.6% identity and 54.2% coverage. TvSom1 was then used to search *T. atroviride*, *reesei,* and *asperellum* genomes (Figure 3).

Phylogeny of the predicted Som1/Flo8 orthologs (Figure 3A) follows taxonomy, with three groups: ascomycete yeasts, basidiomycetes, and filamentous ascomycetes. The characteristic LisH domain (orange box, Figure 3B) is not detected by ProfileScan or Pfam in *T. virens* and *T. reesei*, although homology in the LisH region is strong (alignment, Figure 3B). Nevertheless, the 9-amino-acid transactivation domain which overlaps the LisH domain is detected (https://www.med.muni.cz/9aaTAD/, accessed on 19 December 2021) in these two species. Two NLS are detected by PSORT in all the filamentous ascomycete homologs shown in Figure 3 (in the vicinities of 250 and 640 bp depending on the species); yeast Flo8 has one NLS, at bp 451. Protein models are from JGI Mycocosm [42]. Protein sequences were aligned using MUSCLE at the EBI website. The JGI database model for *T. virens* apparently needs to be extended at both the N and C termini, as inferred from an alignment with the published *V. dahliae* sequence.

#### 2.3.2. Expression of TvSom1 and Adhesin Candidate Genes

**Figure 4:**
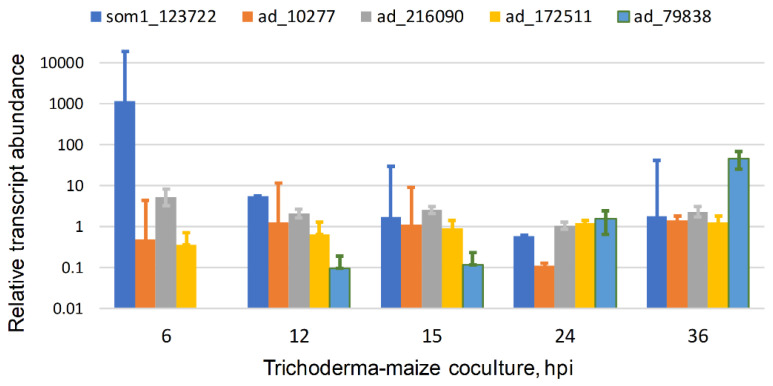
Expression of TvSom1 and four adhesin candidates in interaction with maize roots. RNASeq data are shown for *T. virens* in interaction with maize in hydroponic cultures. Transcript abundance is plotted relative to mycelia in axenic culture, mean, and SEM of three independent samples, from data of [41]. Adhesin candidates are noted as “ad_” and the gene identifiers are JGI *T. virens* v2.0 protein ID numbers. ad_10277 is the GLEYA domain protein (see text), ad_261090 is a Mad2-like protein (Figure 2), and ad_172511 and ad_79838 are Fas1 and Wsc1 homologs, respectively. hpi, hours post-inoculation of Trichoderma germlings.

**Figure 5:**
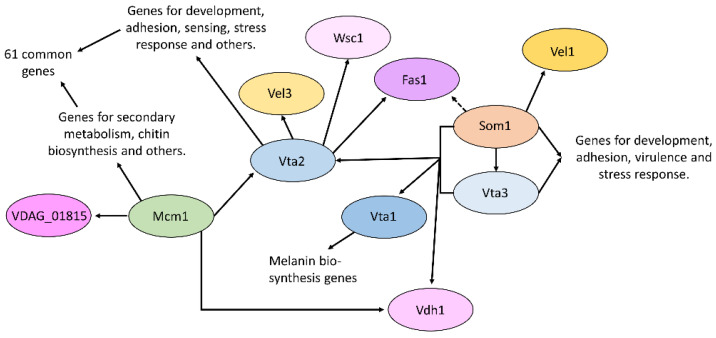
Network of *V. dahliae* transcription factors, which are involved in the regulation of adhesion. Black arrows indicate a transcriptional control which might be either direct or indirect. For example, the dashed line indicates regulation downstream of Som1; however, Vta2 is itself regulated by Som1. A possible Mad2-like adhesin (VDAG_01815) is a target of Mcm1 but not of VTA2. For further details see the main text.

TvSom1 is expressed in interaction with maize roots in hydroponic co-culture. Transcript levels, from RNASeq data [41], were highest at 6 h interaction, decreasing thereafter (Figure 4). The time window for the strong expression of TvSom1 may be narrow because in experiments done under different conditions (B.A.H., unpublished) the transcript level at 6 hpi was similar to that at 30 and 54 h. This will need to be addressed by further short-time series. In these experiments (Figure 4), a suspension of germinated conidia was inoculated into the hydroponic medium; thus, the strong expression at 6 hpi suggests a function for TvSom1 in the early interaction of germlings with the roots. The construction of mutants in TvSom1 may answer the question of whether TvSom1 is required for the expression of the adhesin candidates in Figure 2, or others to be discovered by transcriptomics. Three of the adhesin candidates were expressed in both axenic culture and in interaction with maize, showing overall similar levels over the time course, with the exception of the decrease at 24 h. Any functional significance of the minimum in expression at 24 h (consistent over the three experiments) is not clear. The expression of these three genes does not appear to depend directly on TvSom1, as there was no peak in expression at 6 h. The adhesin candidate 79838 showed increased expression at a longer time in interaction with roots, 36 hpi, when colonization is already extensive. Adhesin candidates 172511 and 79838 are the Fas1 and Wsc1 predicted orthologs mentioned above, respectively. They were not co-regulated with *T. virens* Som1 (123722). Thus, the transcriptomic data (at least those available so far) do not support the regulation of any of the adhesin candidates in *Trichoderma virens* by a Som1-dependent network corresponding to the one elucidated in *V. dahliae* (Figure 5).

Aside from yeast, the Som1/Flo8 coding sequences are interrupted by several introns: one or two in the C terminal part and one or two in the N terminal part. The *V. dahliae* JGI model shown has three predicted introns, while the one from strain JR2 has four in total, with a third one in the N-terminal half, and one in the C-terminal half. *T. virens* and *T. reesei* have two predicted introns in their N terminal part, while *T. atroviride* and *T. asperellum* have one. In *T. virens*, variants for splicing of only i1 or only i2 were detected by sequencing of cDNA (B.A.H., unpublished). The initial quantitation by qPCR with splice variant-specific primers at 16 (mainly germlings) and 24 h (mainly branching small colonies) post-germination in shake culture indicated the expression of both i1-only and i2-only variant transcripts, at the order of 1% of the main species in which both i1 and i2 were spliced. The low expression level made it difficult to ask whether there is any difference between the two time points tested. Intron i1 would not interrupt the reading frame, creating an insertion as compared to the fully spliced model (Figure 3B), while if intron i2 were not spliced, the protein would end at the stop codon located in the start of intron i2. Alternative splicing of the SomA transcript was confirmed in *A. fumigatus* and in this case, the shorter variant was nearly full length and functional. Both the long and short forms complemented non-adherent yeast to the adhering phenotype [31]. The rice blast ortholog MoSom1 has six variants, all of which translate to approximately the same size protein but carry either small insertions or deletions relative to the fully spliced form [46]. These observations of alternative splicing in at least three ascomycete species might suggest some functional significance. Differential functions for splice variants are less prominent in fungi than in the mammalian transcriptome, but are nonetheless known: The transcript of *Neurospora crassa FREQUENCY* (*FRQ*), the core regulator of the circadian clock, is an example [47,48], and this is a growing field [49]. Flo8 orthologs, given their hub position in regulating adhesion and development, could be a good choice for future study.

#### 2.3.3. Transcriptional Network

In Verticillium, six transcription factors were identified that were able to restore adhesion in non-adhesively growing yeast, named Vta1-6 [32]. Vta1-3 has been characterized so far in addition to the *V. dahliae* Som1 transcription factor. Vta3 and Som1 are both required for conidiation, resting structure formation, as well as virulence [14]. Som1 is required for initial root colonization, by contrast, in the absence of Vta3-reduced colonization of the root with few penetration points observed. Both transcription factors control the expression of genes for development, adhesion, and stress response [32,50]. This includes Som1 control of the potential adhesin Fas1 and the velvet domain transcription factor protein Vel1. Velvet proteins are important regulators of fungal development and secondary metabolism [51,52]. Additionally, Som1 and Vta3 control the expression of the hydrophobin Vdh1 and the transcription factor Vta1, which also restored adhesion in yeast. Vta1 seems not to be involved in the adhesion of *V. dahliae*, but instead regulates the formation of melanin, which protects the resting structures [50]. Vta2 is a C2H2 zinc finger transcription factor, which is important for growth and conidiation, and negatively regulates resting structure formation [32]. The protein is not required for the first contact with the host, initial colonization, or penetration, but for further colonization in the root. Vta2 controls the expression of many different genes, which are linked to diverse cellular processes such as development, sensing, and stress response. This also includes a member of the velvet family, Vel3. Vta2 further controls the expression of genes for a number of potential adhesins, such as Fas1 and a Fas1-like protein. The expression of Vta2 in turn is controlled by Som1 as well as Vta3. In addition, the MADS-box transcription factor Mcm1 also controls Vta2 transcript levels [15]. Mcm1 is involved in secondary metabolism and developmental processes. It was also shown to be required for conidia adhesion to the root surface, and plants infected with the corresponding deletion strain showed a reduction in disease symptom development. Transcriptomic data showed that 61 transcripts with altered expression in the absence of Vta2 also showed altered regulation upon deletion of Mcm1 [15]. In addition, Vdh1 also is regulated by Mcm1.

This network could apply to Trichoderma as well, given the similarity in the way the fungal–root interaction starts in these two Sordariomycetes. Experience from the study of fungal signaling pathways, however, suggests that there could be variations, perhaps reflecting the lifestyle of Trichoderma as a mutualist rather than a pathogen. Well-studied pathways from one species, nevertheless, are the best starting point to generalize from. A first hint that some of the molecular machinery for adhesion itself is conserved at the sequence level comes from our identification of VDAG_01815 (Figure 2) as under-expressed in published RNASeq data for the Mcm1 deletion strain [15]. This adhesin candidate is a possible ortholog of *Metarhizium anisopliae* Mad2, which in turn led to predictions for Trichoderma species (Figure 2). Deletions can be constructed in the predicted Trichoderma orthologs of the genes in Figure 5. An initial BLAST search using the corresponding *V. dahliae* JR2 identifiers at EnsemblFungi (https://fungi.ensembl.org/index.html, accessed on 27 February 2022) led to the following well-conserved candidates: VTA2, XP_013954105.1, Mcm1, XP_013958151.1, and VTA3, XP_013961645.1. The best hit for hydrophobin Vdh1 is XP_013956717.1, which is a class II hydrophobin with SignalP. The role of hydrophobins in adherence of Trichoderma needs to be systematically addressed across the different hydrophobin classes, and we note again here the evidence for a *T. asperellum* class I hydrophobin (Figure 1A). Although hydrophobins do not fit the criterion that adhesins are expected to have a binding site at one end and an anchor at the other, they do have a profound influence on cell surface properties of both fungal and, potentially, host cells, along with the ability to form amyloid-like fibrils [53]. Further muddying the waters, a close *T. virens* homolog to TasHyd1, HFB9a, was found to not have a role in the adhesion of the hyphae/germlings to plant roots, but rather to assist in the colonization process post-adhesion (Taylor et al., in preparation). Trichoderma orthologs of Vel1 (Figure 5) are already studied in other developmental contexts [54,55,56,57]. Another way to tackle this question would be a new screen in yeast for the elements of the Trichoderma adhesion program, along with the strategy applied to *A. fumigatus* and *V. longisporum* [14,31]. Even before embarking on such a project, the first priority might be to find out whether adhesion is indeed important for the Trichoderma–plant interaction. Mutants deleted for the Som1 orthologs provide a convenient starting point, if they prove to have defects in adhesion. Another strategy to try would be to add a surfactant/detergent at low concentrations to root–fungal interactions in hydroponic culture to prevent adhesion, and assay for phenotypes in colonization or the induction of systemic resistance of the host plant to pathogen infection.

## 3. Outlook

Knowledge from Verticillium and Metarhizium will facilitate finding the corresponding molecular machinery in Trichoderma spp. Conversely, the different lifestyles allow us to identify the aspects of adhesion that are related to a pathogen or mutualistic lifestyles, or perhaps to show instead that the mechanisms are lifestyle-independent. In a more general context, symbionts other than Trichoderma could share mechanisms of adhesion. AMF mycorrhizae form adhesion structures; however, little is known at the molecular level. Ascomycete sequences may be less helpful in the identification of AMF orthologs, and other genetic screens need to be devised. An initial search of the *Rhizophagus irregularis* genome (DAOM 197198 v2.0, [58]) at JGI Mycocosm [42] with yeast Flo8, for example, detected a single hit with homology, but this was restricted to a small region of the N-terminal part, including a LisH domain. It remains to be seen what the properties of the adhesion program of basal fungal lineages will be.

## Data Availability

All data are provided within the manuscript. The full transcriptomic data set that is the source for Figure 4 is accessible via the citation and at NCBI GEO, accession no: GSE181269.
